# The role of family physicians in emergency and essential surgical care in the district health system in South Africa

**DOI:** 10.4102/safp.v62i1.5117

**Published:** 2020-07-22

**Authors:** Kathryn Chu, Priyanka Naidu, Steve Reid, Hans Hendriks, Jenny Nash, Vanessa Lomas, Francois Coetzee, Robert Mash

**Affiliations:** 1 Centre for Global Surgery, Department of Global Health, Faculty of Medicine and Health Sciences, Stellenbosch University, Cape Town, South Africa; 2 Primary Health Care Directorate, Faculty of Health Sciences, University of Cape Town, Cape Town, South Africa; 3 Department of Family Medicine, Faculty of Health Sciences, Zithulele Hospital and Walter Sisulu University, Zithulele, South Africa; 4 Eastern Cape Department of Health, Amathole District, Eastern Cape, South Africa; 5 Division of Family Medicine and Primary Care, Faculty of Medicine and Health Sciences, Stellenbosch University, Cape Town, South Africa; 6 Department of Global Health, Faculty of Medicine and Health Sciences, Stellenbosch University, Cape Town, South Africa

## Introduction

Five billion people lack access to safe surgical care worldwide and the greatest burden of unmet surgical need lies in low- and middle-income countries.^1^ Improved access to timely, safe and affordable surgical care for all is a global health priority.^2^ National surgical planning has been declared an imperative in South Africa, although little priority has been given to this by government policymakers.^3,4^

### Emergency and essential surgical care at the district hospital

The World Health Organization has identified EESC as key components of universal health coverage and the DH as the first point of access to surgical care.^5^ The World Bank published *Disease Control Priorities*, a textbook which identified 44 cost-effective EESC procedures; 28 of which are recommended as DH procedures.^6^ The bulk of surgical care in South Africa is delivered at regional and tertiary hospitals. However, long waiting times for outpatient appointments for elective conditions and inpatient operative theatre delays for emergency procedures are not uncommon at these facilities.^7^ Historically, surgical care delivery at DHs in South Africa has been limited,^8^ but the decentralisation of treatment for certain surgical conditions could improve access to timely and quality surgical care in the country. This editorial explores the potential role of the family physician (FP) in strengthening decentralised EESC at the DH level. It discusses the importance of establishing an EESC DH package of care and support from higher-level facilities.

### The role of family physicians

**FIGURE 1:**
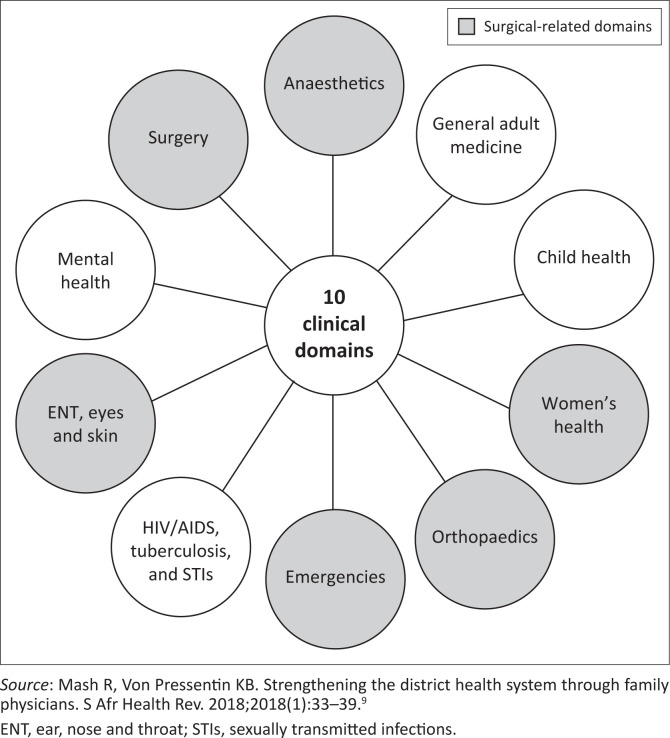
The 10 clinical domains of the family physician at the district hospital.

Family medicine (FM) was recognised as a medical specialty by the Health Professions Council of South Africa in 2007, and nine South African universities currently have FM postgraduate training programmes.^9^ Of the 10 clinical domains outlined for FP training,^9^ six are related to the delivery of surgical care (Figure 1). The South African Academy of Family Physicians and the College of Family Physicians of South Africa have advocated for at least one FP at each of the 244 DHs.^10^ Most FM postgraduate training programmes require training in 18 of the 28 World Bank EESC DH procedures.^11^ A previous study acknowledged the importance of major surgical skills in the scope of FP practice,^12^ and at a workshop at the 2019 Rural Health Conference, South African FPs also expressed interest in improving access to safe and timely surgical care at DHs. Several barriers were identified as obstacles to scaling up DH EESC by FPs at the workshop, including staff shortages, insufficient skills mix in the DH, a lack of support from surgical and anaesthetic departments at higher levels of care, a lack of funding for equipment and supplies, and a lack of appropriate post-operative care.^8,13,14^

Family physicians are uniquely poised to champion decentralised surgical services for several reasons. They are taught to spearhead DHs’ clinical governance and to act as ‘change agents in the system, offering significant leadership to help take the health services forward’.^10^ This means that they have a responsibility for access to, and the quality of, surgical services in their districts, including the training and supervision of junior staff. In addition, they are mandated to work with specialists and subspecialists at higher-level hospitals.^15,16^

Districts may differ – for example, large DHs in metropolitan areas may offer services similar to a regional hospital with surgical departments and some districts may have easy access to a regional hospital, while other districts are very remote. To effectively plan the surgical services in a health district or region, it is necessary to map the available resources (human and physical), not by facility silos but as an integrated health system. This assessment would lead to a better understanding of what procedures can be performed safely and what inputs in terms of workforce, support, equipment and supplies are needed to provide the intended package of care. This, however, can only be implemented with support from the regional- and tertiary-level hospital surgeons and anaesthetists. Such support could include outreach, training, mobile health referral applications and discussion groups, and improved referral and transfer systems.

## Conclusion

In summary, we need to do the following to strengthen DH surgical services:

- Update the package of emergency and essential surgical procedures for the DH.
- Ensure the appropriate equipment and an adequate supply chain for surgical care.
- Employ FPs at DHs to strengthen the ability to deliver surgical care and anaesthesia as well as to provide the needed clinical leadership and governance.
- Enlist support from surgeons and anaesthetists at the regional and tertiary hospitals.

Strengthening DH surgical services would improve universal health coverage, an important objective of the upcoming National Health Insurance scheme. FPs who are the cornerstone DH cadre have surgical and anaesthetic technical skills and leadership training and can play a pivotal role.
